# *VCAM-1* predicts poor prognosis and modulates immune infiltration in gastric cancer: a TCGA-based bioinformatics study

**DOI:** 10.3389/fgene.2025.1602929

**Published:** 2025-08-25

**Authors:** Cheng Wu, Yungeng Liu, Chuanyuan Liu, Chuanfa Fang

**Affiliations:** ^1^ Department of Gastrointestinal and Hernia Surgery, Ganzhou Hospital-Nanfang Hospital, Southern Medical University, Ganzhou, China; ^2^ Department of Gastrointestinal and Hernia Surgery, The Affiliated Ganzhou Hospital of Nanchang University, Ganzhou, China

**Keywords:** *VCAM-1*, gastric cancer, prognostic biomarker, immune infiltration, TCGA database

## Abstract

**Background:**

Gastric cancer (GC) is a leading cause of cancer-related mortality; however, biomarkers predicting its immunotherapy resistance remain scarce. Vascular cell adhesion molecule (*VCAM*)-*1*, an immune cell adhesion mediator, is implicated in tumor progression; however, its prognostic and immunomodulatory roles in GC remain unclear.

**Methods:**

In this study, we analyzed *VCAM-1* expression and its clinical relevance in GC using RNA-sequencing data from The Cancer Genome Atlas. Differential gene analysis, gene set enrichment analysis (GSEA), and single-sample GSEA were used to identify the underlying pathways and immune infiltration patterns. Validation was performed via Cox regression, receiver operating characteristic, and immunohistochemical (Human Protein Atlas database) analyses.

**Results:**

*VCAM-1* expression levels were significantly upregulated in the GC tissues (p < 0.001) and correlated with advanced T stage (p = 0.046), N stage (p = 0.047), and poor overall survival (hazard ratio = 1.54; p = 0.046). GSEA linked *VCAM-1* expression to various immune pathways (e.g., interleukin-17 signaling), and single-sample GSEA revealed its positive associations with the Th1, cytotoxic, and CD8^+^ T cell proportions (p < 0.05) and inverse correlation with the Th17 cell proportion. Immunohistochemistry revealed elevated VCAM-1 protein levels in the tumors.

**Conclusion:**

*VCAM-1* is a novel prognostic biomarker driving immunosuppressive microenvironmental remodeling in GC. Furthermore, its dual roles in immune regulation highlight its potential to optimize GC immunotherapy.

## Introduction

Gastric cancer (GC), a prevalent digestive system malignancy, is the leading cause of cancer-related morbidity and mortality worldwide (Bray et al., 2024). Recently, clinical management of GC has significantly improved, primarily due to the introduction and application of immune checkpoint blockade therapies (Lu et al., 2022). These therapies target the key immune checkpoints, specifically programmed cell death protein 1 and cytotoxic T-lymphocyte-associated protein 4, to rejuvenate the activity and potentiate the antitumor effects of T cells (Wang et al., 2022). Despite recent advancements, therapeutic benefits remain limited and are primarily observed in only some patients. Unfortunately, a significant proportion of patients exhibit immune tolerance and disease progression after treatment, highlighting the urgent need to elucidate the underlying immune regulatory mechanisms and identify the key targets in GC. Further exploration is crucial for the development of effective therapeutic strategies to improve the patient outcomes.

Initially characterized in 1989 for its vital role in mediating cell adhesion in melanoma (Osborn et al., 1989; Rice and Bevilacqua, 1989), vascular cell adhesion molecule (*VCAM*)-*1* has garnered significant attention as a crucial factor for the development and progression of solid tumors, particularly breast cancer and GC (Chen et al., 2011; Kuai et al., 2012). *VCAM-1* is a prominent component of metastasis-specific gene signatures in breast cancer, playing a crucial role in modulating the metastatic processes (Minn et al., 2005). Abnormal *VCAM-1* expression promotes lung and bone metastases in breast cancer (Chen et al., 2011).

*VCAM-1* interacts with integrin VLA-4 in monocytes and tumor-associated macrophages, recruiting it to the lung tissues. Tumor-associated macrophages and monocytes protect the tumor cells by facilitating immune evasion. Macrophages accumulate around the tumor cells through the binding of VLA-4 to homologous *VCAM-1* (Gil-Bernabe et al., 2012; Qian and Pollard, 2010). Wu et al. reported that *VCAM-1* expression correlates with increased immune resistance in renal cancer (Wu et al., 1995). Furthermore, *VCAM-1* binding to VLA-4 on their surfaces triggers T cell migration (Rose et al., 2002).

Currently, the complex relationship between *VCAM-1* expression and immune cell infiltration and its effects on the patient overall survival (OS) and tumor infiltration patterns remain unknown. To bridge this knowledge gap, we used bioinformatics techniques to examine the *VCAM-1* expression patterns in GC cells and tissues. Our analysis revealed that high *VCAM-1* levels were correlated with the patient clinicopathological characteristics and prognosis in GC. Our results also revealed the correlation between elevated *VCAM-1* levels and immune infiltration, providing novel insights into the intricate interplay between *VCAM-1* and the GC immune microenvironment. Our findings can aid in the identification of novel therapeutic targets and improvement of clinical outcomes in patients with GC.

## Methods

### Data acquisition and preliminary analysis

RNA sequencing data (The Cancer Genome Atlas [TCGA]–stomach adenocarcinoma cohort; n = 375 tumor and 32 normal samples) in TPM format were obtained from TCGA database (https://portal.gdc.cancer.gov/), which covers 33 diverse cancer types. The data were subjected to rigorous preprocessing, particularly focusing on the *VCAM-1* expression profiles. Subsequently, Wilcoxon rank-sum test was performed to assess the *VCAM-1* expression changes using the built-in stats package within the R statistical software (version 4.2.1) environment. Moreover, visualization capabilities of ggplot2 (version 3.3.6) were used to create intricate graphical representations highlighting the complex *VCAM-1* expression patterns across different tumor types.

### Differentially expressed gene analysis

HTSeq-count samples were divided into high and low expression groups using the median *VCAM-1* level as the cut-off. Patients were stratified into the high and low *VCAM-1* expression groups based on the median *VCAM-1* expression level, which is a commonly used threshold in transcriptomic studies for a balanced group size and clinical relevance (van Hoorn et al., 2017). Subsequent analysis was performed using DESeq2 (version 1.36.0) and edgeR (version 3.38.2), with statistical significance determined by an adjusted p-value <0.05 and absolute log2-fold change (|log2FC|) > 1.5, which served as thresholds to identify DEGs.

### Enrichment analysis

Functional enrichment analysis was performed using the ClusterProfiler package in R (version 4.4.4) to identify the Gene Ontology (GO) terms, followed by gene set enrichment analysis (GSEA). DEGs were selected based on *VCAM-1* expression. GO analysis involves three main categories: Cellular components, molecular functions, and biological processes. GSEA is a computational method evaluating the statistical significance and consistency of gene expression changes for a specified gene set under two biological conditions. The normalized enrichment score and adjusted p-value were used as the criteria to prioritize the enriched pathways for each phenotype. Gene sets with false discovery rates <0.25 and adjusted p-values <0.05 were considered to be significantly enriched.

### Immune infiltration analysis

Using the GSVA package (version 1.46.0) (Hanzelmann et al., 2013) in R, single-sample GSEA was conducted to explore the relationship between *VCAM-1* and the signature genes in 24 immune cell types. We systematically analyzed the immune infiltrates associated with *VCAM-1* using published literature (Bindea et al., 2013). Both Spearman’s correlation and Wilcoxon rank-sum tests were used to compare immune cell infiltration between the *VCAM-1* high and low expression groups.

### Protein–protein interaction network

Next, protein–protein interaction network of coregulated DEGs and functional interactions among proteins were analyzed using the Search Tool for the Retrieval of Interacting Genes/Proteins database (http://string-db.org) (Szklarczyk et al., 2019). The results were visualized using the Cytoscape software.

### Validation analysis

Immunohistochemistry data from the Human Protein Atlas database (https://www.proteinatlas.org/) were analyzed to further validate our findings. This analysis provided visual evidence of the *VCAM-1* expression patterns in the normal and tumor tissues.

### Statistical analyses

Statistical data from TCGA database were processed using R (version 4.2.1). Wilcoxon rank-sum and signed-rank tests were used to compare the *VCAM-1* expression levels between patients with GC and healthy controls. Associations between *VCAM-1* levels and various clinicopathological factors were assessed via Welch’s one-way analysis of variance, followed by the Bonferroni correction or t-test, as appropriate. Hazard ratio (HR) for OS was estimated using univariate Cox proportional hazard regression models, whereas HR for individual factors was determined using 95% confidence intervals. Receiver operating characteristic (ROC) analysis of *VCAM-1* was performed using the pROC package, yielding an area under the curve of 0.5–1.0, indicating 50%–100% discrimination ability. Time-dependent ROC curve analysis was conducted to assess the ability of *VCAM-1* to predict the GC outcomes at one, three, and 5 years. ROC analysis for VCAM-1 diagnostic accuracy estimation (tumor vs. normal) and time-dependent ROC analysis for survival prediction were performed using TCGA–stomach adenocarcinoma cohort. All statistical tests were considered significant at two-tailed p-values ≤0.05.

## Results

### Clinical characteristics

**FIGURE 1:**
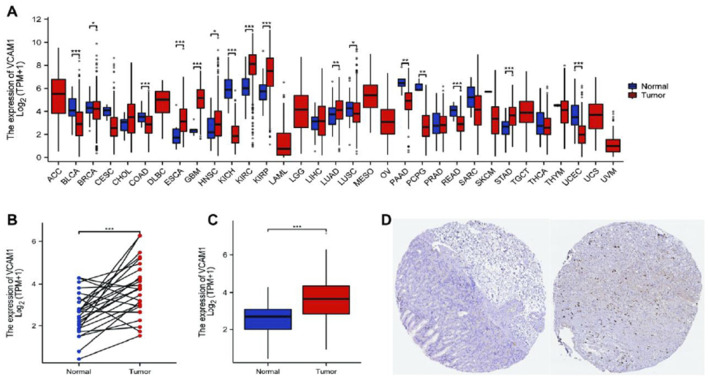
Expression level of *VCAM-1* gene in GC. **(A)** *VCAM1* expression (log2 (TPM+1)) across TCGA cancer types (T) vs. normal tissues (N). Red: Tumor, Blue: Normal. Wilcoxon rank-sum test. **(B)** *VCAM1* expression in GC tumors vs. paired adjacent normal tissues (n = 27 pairs). Paired Wilcoxon test, p < 0.01. **(C)** *VCAM1* expression in unpaired GC tumors vs. normal tissues. Wilcoxon rank-sum test. **(D)** Representative IHC images from HPA database showing *VCAM-1* protein expression (brown stain) in normal gastric tissue (negative) and GC tissue (medium/strong). *P < 0.05; **P < 0.01; ***P < 0.001.

Using TCGA database, our analysis of *VCAM-1* expression patterns in various cancer and normal tissues revealed the notable upregulation of *VCAM-1* levels in most cancer types (Figure 1A). Specifically, both unpaired and paired differential expression analyses of the normal and GC cohorts revealed significantly higher *VCAM-1* levels in the tumor tissues than in the normal tissues (Figures 1B,C). Clinical characteristics, including age, sex, TNM staging, pathological stage, histological grade, and survival, of 375 patients with GC are summarized in Table 1. The study cohort consisted of 241 males and 134 females. Fisher’s exact test revealed a significant correlation between *VCAM-1* expression and patient OS (p = 0.029). Additionally, chi-square analysis indicated the correlations between *VCAM-1* expression and pathological stage (p < 0.001), T stage (p = 0.046), N stage (p = 0.074, approaching significance), and histological grade (p < 0.001), with no significant correlation observed with the other clinicopathological features. To further explore the *VCAM-1* expression patterns in tumors, we analyzed immunohistochemistry data from the Human Protein Atlas database. Notably, normal tissues exhibited negative *VCAM-1* staining, in contrast to tumor tissues, which exhibited medium-to-strong *VCAM-1* staining. This suggests that *VCAM-1* expression levels are upregulated in malignancies (Figure 1D). These findings suggest *VCAM-1* as a potential biomarker and therapeutic target for various cancer types, including GC.

**TABLE 1:** Demographic and clinicopathological parameters of high and low VCAM-1 expression group patients with GC in TCGA.

Characteristics	Low expression of VCAM1	High expression of VCAM1	P value
n	187	188	
Gender, n (%)			0.695
Male	122 (32.5%)	119 (31.7%)	
Female	65 (17.3%)	69 (18.4%)	
Age, n (%)			0.318
≤ 65	77 (20.8%)	87 (23.5%)	
>65	108 (29.1%)	99 (26.7%)	
Pathologic stage, n (%)			<0.001
Stage I	41 (11.6%)	12 (3.4%)	
Stage II	48 (13.6%)	63 (17.9%)	
Stage III	73 (20.7%)	77 (21.9%)	
Stage IV	17 (4.8%)	21 (6%)	
Pathologic T stage, n (%)			<0.001
T1&T2	65 (17.7%)	34 (9.3%)	
T3&T4	121 (33%)	147 (40.1%)	
Pathologic N stage, n (%)			0.046
N0	65 (18.2%)	46 (12.9%)	
N1&N2&N3	116 (32.5%)	130 (36.4%)	
Pathologic M stage, n (%)			0.563
M0	165 (46.5%)	165 (46.5%)	
M1	11 (3.1%)	14 (3.9%)	
Histologic grade, n (%)			<0.001
G1	6 (1.6%)	4 (1.1%)	
G2	92 (25.1%)	45 (12.3%)	
G3	84 (23%)	135 (36.9%)	
OS event, n (%)			0.029
Alive	124 (33.1%)	104 (27.7%)	
Dead	63 (16.8%)	84 (22.4%)	

### Associations between *VCAM-1* expression and various clinicopathologic variables

**FIGURE 2:**
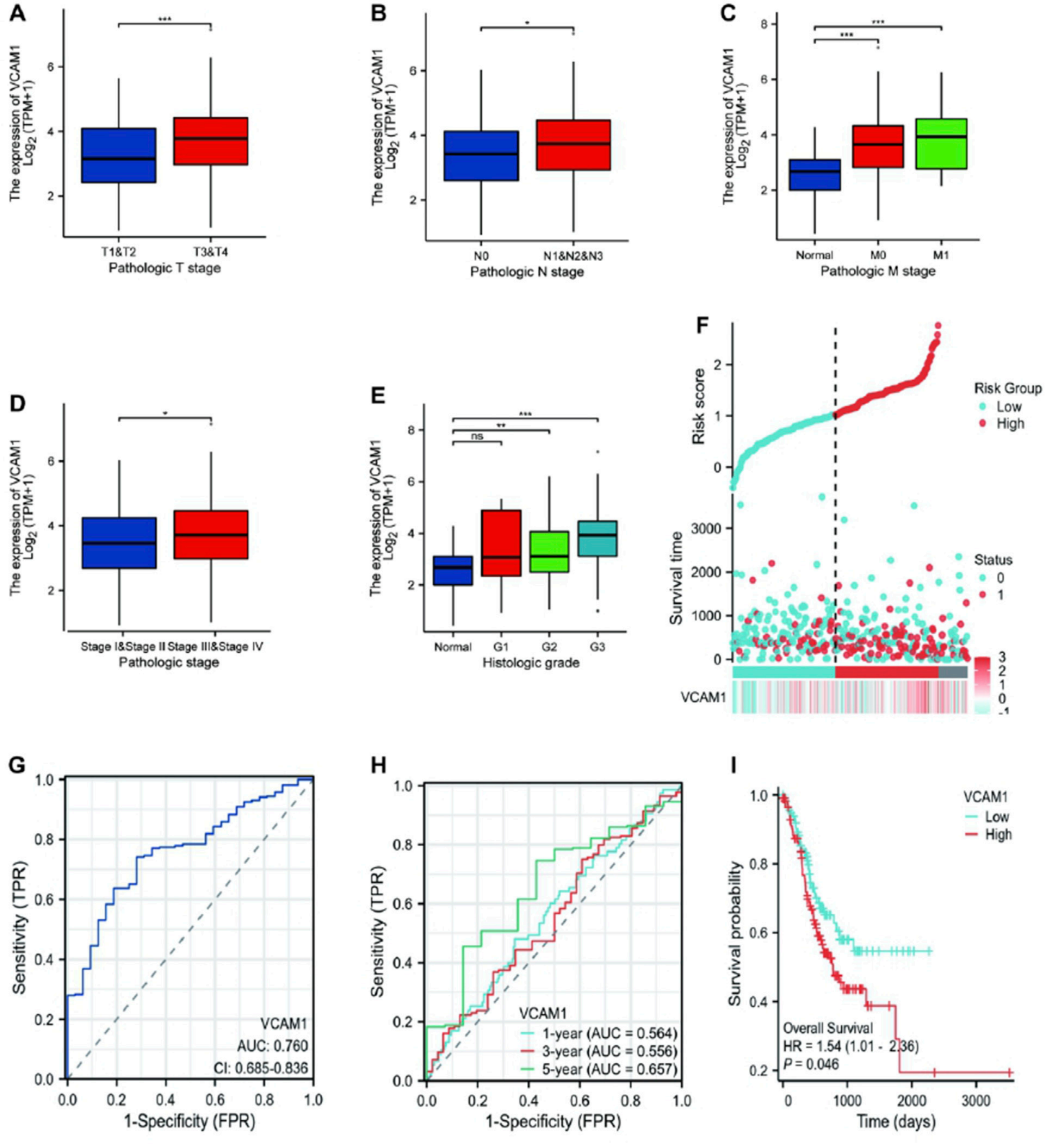
Association between the *VCAM-1* expression and different clinicopathologic characteristics. **(A–E)** Boxplots showing *VCAM1* expression (log2 (TPM+1)) across **(A)** T stages, **(B)** N stages, **(C)** M stages, **(D)** Pathologic stages, **(E)** Histologic grades. Welch’s ANOVA or t-test p-values shown. **(F)** Distribution of *VCAM1* expression (ordered left to right), patient survival status (Blue dots: inceased patients; Red dots: deceased patients), and risk score. Vertical dashed line: Median risk score separating Low (Left) and High (Right) risk groups. **(G)** ROC curve for *VCAM1* diagnostic power (Tumor vs. Normal). AUC and 95% CI shown. **(H)** Time-dependent ROC curves for *VCAM1* predicting 1-, 3-, and 5-year overall survival. AUC values shown. **(I)** Kaplan-Meier survival curves for High vs. Low *VCAM1* expression groups. Log-rank p-value and Hazard Ratio (HR) with 95% CI shown.

Welch’s one-way analysis of variance revealed significant correlations between *VCAM-1* expression and both the M stage and histologic grade of GC (Figures 2C,E). Additionally, t-tests confirmed the significant correlations between *VCAM-1* expression and T, N, and overall pathological stages (Figures 2A,B,D). Logistic regression analysis reinforced these associations, indicating *VCAM-1* as a crucial predictor of the T stage (p < 0.001), N stage (p = 0.047), and histological grade (p < 0.001; Table 2). Figure 2F shows the *VCAM-1* expression distribution, survival status of patients with GC, and corresponding risk scores. Blue dots indicate the increased patients with GC, whereas red dots indicate the deceased patients with GC. Horizontal line indicates the median risk score, clearly separating the low-risk (left, low *VCAM-1* expression) and high-risk (right, high *VCAM-1* expression) groups. As the risk score increased, a notable trend emerged, with an incremental increase in the deceased patient (red dots) proportion, indicating poor survival outcomes and high mortality risk in the high-risk group. To validate the diagnostic accuracy of *VCAM-1*, we performed ROC analysis, which yielded an area under the curve of 0.76 (95% confidence interval: 0.685–0.836; Figure 2G). Additionally, we conducted time-dependent ROC analysis to evaluate the predictive capacity of *VCAM-1* for OS at one, three, and 5 years (Figure 2H). Based on the median *VCAM-1* level, patients with GC were divided into the high and low *VCAM-1* expression groups, with the high expression group showing a strong association with poor OS, as indicated by an HR of 1.54 (95% confidence interval: 1.01–2.36; p = 0.046) in Figure 2I. These results confirm the clinical importance of *VCAM-1* as a promising biomarker for the risk stratification and prognostic prediction of GC.

**TABLE 2:** VCAM-1 expression correlated with clinicopathological characteristics analyzed by logistic regression.

Characteristics	Total (N)	OR (95% CI)	P value
Pathologic T stage (T3&T4 vs. T1&T2)	367	2.323 (1.438–3.752)	<0.001
Pathologic N stage (N1&N2&N3 vs. N0)	357	1.584 (1.007–2.491)	0.047
Pathologic M stage (M1 vs. M0)	355	1.273 (0.561–2.886)	0.564
Pathologic stage (Stage IV&Stage III vs. Stage I&Stage II)	352	1.292 (0.849–1.966)	0.231
Histologic grade (G3 vs. G1&G2)	366	3.214 (2.074–4.981)	<0.001

### Association between *VCAM-1* expression and immune infiltration

**FIGURE 3:**
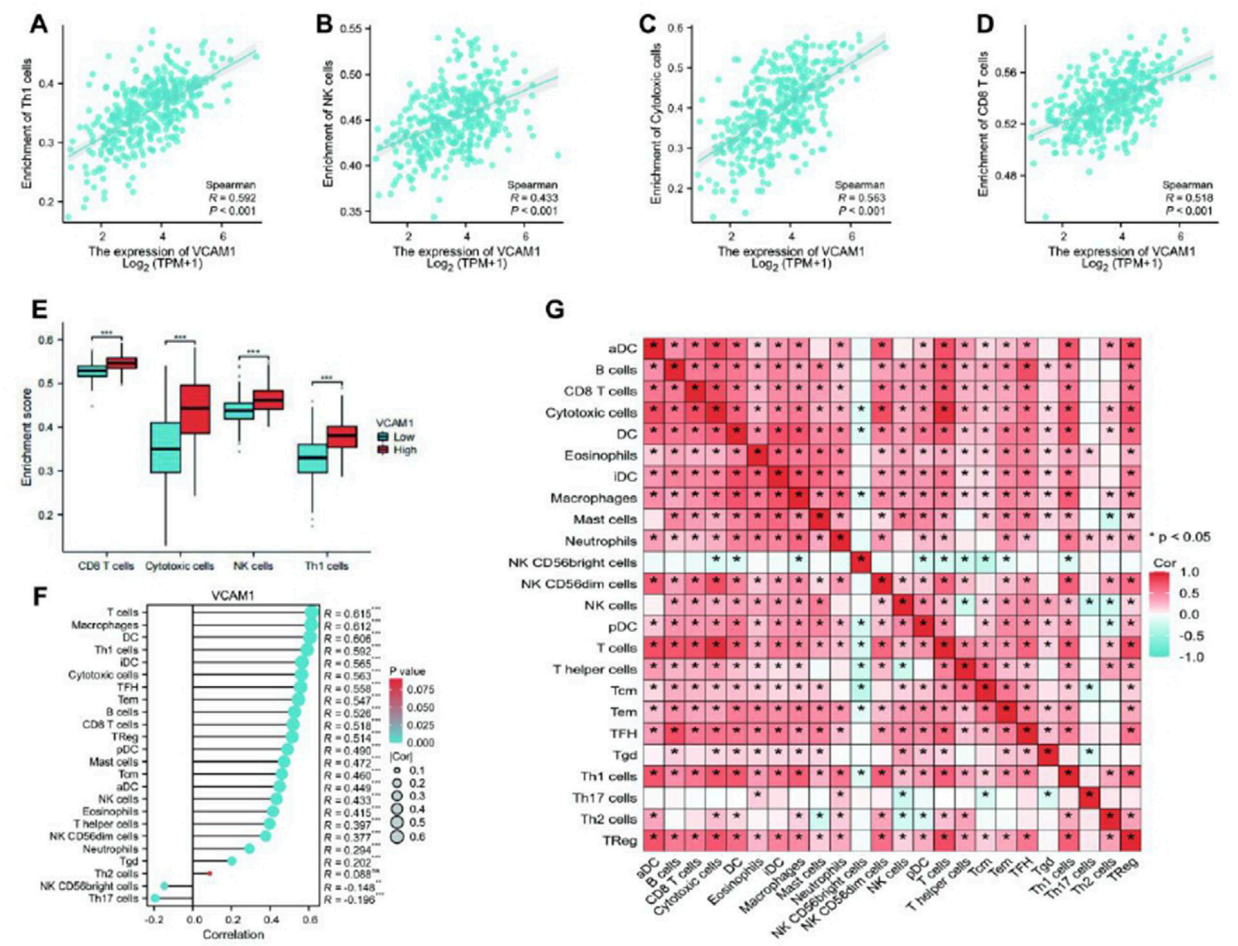
The results of analysis between *VCAM-1* expression and immune infiltration. **(A–D)** Scatter plots showing Spearman correlation (r and p-value) between *VCAM1* expression (log2 (TPM+1)) and SSGSEA scores for **(A)** Th1 cells, **(B)** NK cells, **(C)** Cytotoxic cells, **(D)** CD8^+^ T cells. **(E)** Boxplots comparing ssGSEA scores of key immune cells between High and Low *VCAM1* groups (Wilcoxon rank-sum test, *p < 0.05). **(F)** Bar plot showing Spearman correlation coefficients (r) between *VCAM1* expression and ssGSEA scores of 24 immune cell types. *p < 0.05. **(G)** Heatmap of Spearman correlation coefficients among ssGSEA scores of 24 immune cell types in GC samples. Color scale: Red (positive), Blue (negative).

Spearman’s correlation analysis was conducted to examine the association between *VCAM-1* expression (in TPM format) and immune cell infiltration quantified using the single-sample GSEA scores. Our results revealed that *VCAM-1* expression was positively correlated with Th1, natural killer (NK), and cytotoxic and CD8^+^ T cell infiltration (Figures 3A–D). Notably, infiltration of these immune cell subsets was significantly higher in the *VCAM-1* high expression group than in the *VCAM-1* low expression group (p < 0.05; Figure 3E). *VCAM-1* expression was also positively correlated with the infiltration of multiple immune cell types, including T cells, macrophages, dendritic cells (immature and activated), T follicular helper cells, effector memory T cells, B cells, regulatory T cells, plasmacytoid dendritic cells, mast cells, and central memory T cells. In contrast, an inverse correlation was observed between *VCAM-1* expression and NK CD56bright and Th17 cell infiltration (Figure 3F). These results suggest the crucial role of *VCAM-1* in regulating the immune infiltration patterns in GC. A heatmap was plotted to further understand the complexity of the correlations among the 24 distinct tumor-infiltrating immune cell subpopulations (Figure 3G). The heat map provided a comprehensive snapshot of the varying degrees of correlation among different immune cell subsets, revealing intricate patterns potentially affecting the immunobiology of GC.

### Differential expression analysis of *VCAM-1* in GC

**FIGURE 4:**
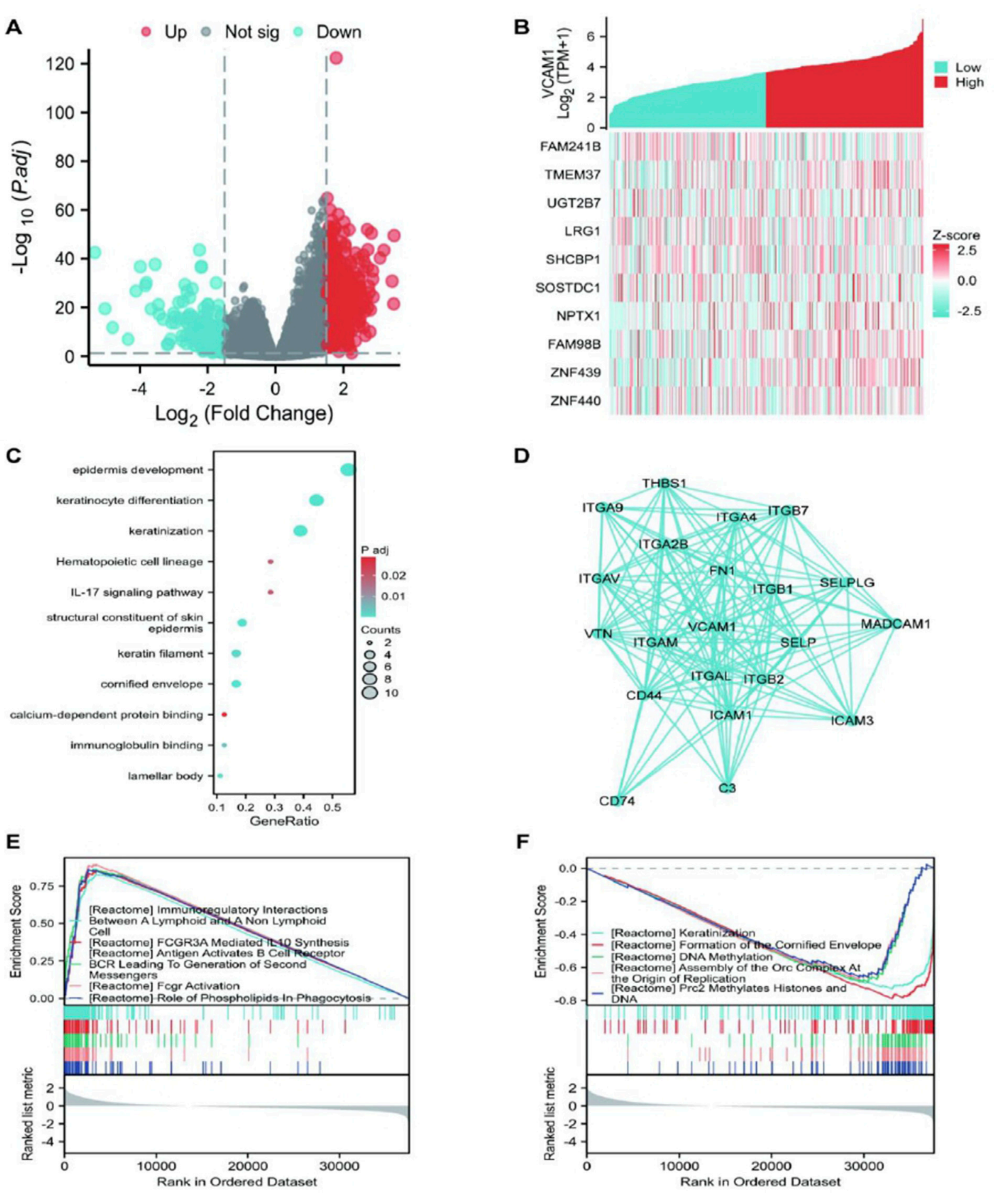
The results of differentially expressed gene (DEG) analysis and Enrichment analysis of *VCAM-1* gene in GC. **(A)** Volcano plot of DEGs (|log2FC|> 1.5, adj. p < 0.05). Red dots: Significantly upregulated genes; Blue dots: Significantly downregulated genes; Gray dots: Non-significant genes. Dashed lines indicate cutoffs. **(B)** Heatmap showing expression (Z-score) of the top 10 genes positively (Red) and negatively (Blue) correlated with *VCAM1*. **(C)** Top enriched GO Biological Process terms for *VCAM1*-associated DEGs. Dot size represents gene count, color represents adjusted p-value. **(D)** PPI network of top *VCAM1*-interacting partners from STRING database (confidence score >0.7). **(E,F)** Top enriched KEGG pathways from GSEA in High vs. Low *VCAM1* groups. NES: Normalized Enrichment Score, FDR: False Discovery Rate.

Using stringent cut-off criteria of |log2FC| > 1.5 and adjusted p-value <0.05, we analyzed the HTSeq-count data from TCGA, identifying a cohort of 875 DEGs related to *VCAM-1*. DEG expression patterns were visualized using a volcano plot that provided a clear overview of their significance and magnitude of change (Figure 4A). To evaluate potential correlations, the top 5 genes positively or negatively correlated with *VCAM-1* were selected for co-expression heatmap visualization (Figure 4B). In terms of functional implications, GO analysis revealed the significant regulatory roles of *VCAM-1*-associated DEGs in various biological processes, including epidermis development, keratinization, keratinocyte differentiation, epidermal cell differentiation, skin development, immunoglobulin binding, calcium-dependent protein binding, and interleukin-17 signaling. Notably, these DEGs were also observed in the structural components of the epidermis, cytoskeleton, and hematopoietic cell lineages, highlighting their diverse functions (Figure 4C). To visualize the complex network of *VCAM-1* and its co-expression partners among the identified *VCAM-1*-related DEGs, a network diagram was created to provide insights into their potential interactions and functional modules (Figure 4D). To uncover the specific biological functions of *VCAM-1* at the pathway level, we performed GSEA of GC datasets stratified by low and high *VCAM-1* expression. This analysis revealed the Kyoto Encyclopedia of Genes and Genomes pathways significantly associated with *VCAM-1*, providing valuable insights into its potential action mechanisms (Figures 4E,F). These findings highlight the significance of *VCAM-1* in GC, facilitating its further functional validation and therapeutic exploration.

## Discussion

Immune checkpoint blockade therapy has significantly advanced GC treatment (Patel et al., 2022). However, it faces various challenges, such as immunotherapy resistance and immune evasion (Zhang et al., 2021), necessitating the identification of new biomarkers to predict the immunotherapy efficacy. Therefore, in this study, we investigated *VCAM-1* expression in GC and its correlation with patient prognosis using bioinformatics tools. We also analyzed the *VCAM-1* regulation patterns in immune cells within the GC tumor microenvironment (TME). Our findings suggest *VCAM-1* as a potential prognostic predictor for patients with GC and immune infiltration.

In this study, *VCAM-1* levels were significantly upregulated in the GC tissues, consistent with a previous report on *VCAM-1* overexpression in various cancer types (Schlesinger and Bendas, 2015). This study revealed the strong correlation between high *VCAM-1* expression and poor patient prognosis. Expression of *VCAM-1*, a glycoprotein, is induced by various factors, including the tumor necrosis factor-α, reactive oxygen species, and oxidized low-density lipoproteins (Cook-Mills et al., 2011). *VCAM-1* silencing inhibits cancer cell proliferation and is associated with poor prognosis in GC patients (Li et al., 2023). Notably, *VCAM-1* is a promising candidate to improve GC prognosis and treatment.

Using differential gene expression analysis, we identified a set of key tumor-associated genes, including *TMEM37, UGT2B7, LRG1*, and *SHCBP1*. *TMEM37* is an independent prognostic marker for colon cancer (Li et al., 2018). *UGT2B7* activation disrupts the estrogen homeostasis in breasts, exacerbating breast tumor metastasis (Xu et al., 2024). Moreover, activation of the angiogenic factor, *LRG1*, promotes colorectal cancer progression (Zhu et al., 2023). *SHCBP1* knockdown significantly inhibits the proliferation and migration of pancreatic cancer cells *in vitro* (Ma et al., 2024). These findings underscore the crucial role of *VCAM-1* in the regulation of various tumor-related genes, ultimately influencing tumor formation and progression.

In addition to evaluating the prognostic importance of *VCAM-1*, this study explored its complex interplay with the TME, focusing specifically on its strong correlation with immune cell infiltration. TME, encompassing tumor-associated fibroblasts, immune cells, angiogenic cells, and the extracellular matrix, is a vital site for GC cell growth and metastasis. Intercellular interactions and cytokines secreted within the TME collectively contribute to GC progression and immune evasion (Yu et al., 2007). For example, increased B7-H1 (programmed death-ligand 1) expression on GC cell surface inhibits T cell activity, enabling tumor cells to evade immune surveillance (Liu et al., 2022). Furthermore, GC cells promote immune evasion by secreting immunosuppressive factors, such as the transforming growth factor-β and interleukin-10, thereby affecting the immune cell functions in the TME (Yu et al., 2007). This study revealed the strong correlation between elevated *VCAM-1* expression and immune cell (including Th1, NK, and cytotoxic and CD8^+^ T cells) infiltration in GC tissues (Figures 3A–G). Th1 cells are vital immune cells facilitating the elimination of cancer cells via innate immune effectors, such as cytotoxic T lymphocytes, NK cells, and macrophages (Gao et al., 2021). NK cells, a subset of innate lymphoid cells, exhibit diverse killing mechanisms and have recently garnered attention for potential immunotherapy applications (Zhang et al., 2023). Upon entering tumor sites, NK–tumor interactions trigger comprehensive activation signals for various killing activities, including cytolytic granule release, death receptor–ligand interactions, and antibody-dependent cellular cytotoxicity (Huntington et al., 2020). CD8^+^ T cells specifically target and kill tumor cells, playing crucial roles in various cancers (van der Leun et al., 2020).

*VCAM-1* activates the AKT–mechanistic target of rapamycin kinase pathway, which mediates the C-X-C motif chemokine ligand 1 expression and promotes human GC-derived mesenchymal stem cell recruitment, thereby increasing immunosuppression and GC progression (Zhao et al., 2024). This pathway explains the correlation between high *VCAM-1* expression and poor patient prognosis and altered immune infiltration observed in this study. *VCAM-1*-mediated VLA-4 binding recruits tumor-associated macrophages (e.g., in breast cancer metastases to the lungs (Chen et al., 2011)). A negative correlation was observed between *VCAM-1* expression and Th17 cell infiltration (p < 0.05; Figure 3F) in this study. The balance between Th17 and regulatory T cell proportions is an important factor regulating autoimmunity and cancer (Knochelmann et al., 2018). This GC-specific Th17 association possibly contributes to an aggressive phenotype and limited immunotherapy responses in patients with high *VCAM-1* expression.

*VCAM-1* potentially plays active roles in recruiting and activating specific immune cell populations, particularly T cells and macrophages, which subsequently shape the antitumor immune landscape. The complex relationship between *VCAM-1* expression and immune cell infiltration highlights the multifunctional role of this adhesion molecule in gastric cells. *VCAM-1* facilitated the influx of immune cells into the tumor site, potentially orchestrating an immune response to promote or inhibit tumor progression, depending on the context and specific immune cell subsets involved. Therefore, elucidation of the precise mechanisms governing *VCAM-1*-mediated immune cell recruitment and activation is important for the development of novel therapeutic strategies to modulate the TME and enhance the antitumor immunity.

This study has some limitations. First, although the crucial role of *VCAM-1* in GC tumorigenesis was identified, further *in vitro* and *in vivo* experiments are necessary to validate the correlation between *VCAM-1* expression and GC progression and elucidate the fundamental action mechanisms of *VCAM-1* in driving GC progression. Second, clinical studies are vital to assess the associations between *VCAM-1* expression and various clinical characteristics, including tumor staging and prognostic significance, of patients with GC. These evaluations are crucial to gain deeper insights into the ways in which *VCAM-1* expression patterns can guide patient stratification and inform treatment decisions to improve the patient outcomes.

In summary, this study highlighted the significance of *VCAM-1* as a prognostic biomarker and pivotal regulator of immune cell infiltration and TME dynamics in GC. Future studies should explore the molecular mechanisms underlying *VCAM-1* immunomodulatory effects and assess its therapeutic potential for GC. These efforts can contribute to the development of innovative diagnostic, prognostic, and therapeutic approaches for this devastating disease.

## Data Availability

The datasets presented in this study can be found in online repositories. The names of the repository/repositories and accession number(s) can be found in the article/supplementary material.
